# Supplementary material for: Mechanisms governing the pioneering and redistribution capabilities of the non-classical pioneer PU.1
Source: Nat Commun. 2020 Jan 21;11:402. doi: 10.1038/s41467-019-13960-2 (PMC6972792; doi:10.1038/s41467-019-13960-2)
Supplement: Supplementary file 7 — Source data [file 41467_2019_13960_MOESM7_ESM.zip › Source_Data/FigureS5/FigureS5A_MotifsCommonPeaks/homerResults/motif26.similar.html]

motif26

## Information for motif26

C
T
A
G
C
T
A
G
A
C
T
G
T
A
C
G
T
A
C
G
T
A
C
G
T
C
A
G
C
T
A
G
A
T
C
G
  
Reverse Opposite:  

T
A
G
C
A
G
T
C
A
G
T
C
A
T
G
C
A
T
G
C
A
T
G
C
A
G
T
C
A
G
T
C
G
A
T
C
  

|  |  |
| --- | --- |
| p-value: | 1e-57 |
| log p-value: | -1.326e+02 |
| Information Content per bp: | 1.665 |
| Number of Target Sequences with motif | 2102.0 |
| Percentage of Target Sequences with motif | 23.66% |
| Number of Background Sequences with motif | 6165.4 |
| Percentage of Background Sequences with motif | 16.22% |
| Average Position of motif in Targets | 98.2 +/- 56.4bp |
| Average Position of motif in Background | 98.9 +/- 64.6bp |
| Strand Bias (log2 ratio + to - strand density) | -0.1 |
| Multiplicity (# of sites on avg that occur together) | 1.31 |
| Motif File: | file (matrix) reverse opposite |

### Similar de novo motifs found

|  |  |  |  |  |  |  |  |
| --- | --- | --- | --- | --- | --- | --- | --- |
| Rank | Match Score | Redundant Motif | P-value | log P-value | % of Targets | % of Background | Motif file |
| 1 | 0.815 | C T A G T C A G A T C G G T A C C A T G C A T G C T A G T A C G T G A C G A T C C T G A T C G A | 1e-44 | -101.773986 | 10.76% | 6.29% | motif file (matrix) |
| 2 | 0.790 | C A T G A C T G A C T G G T A C C A T G A C T G C T G A C A T G A T G C G A T C | 1e-39 | -91.155676 | 7.83% | 4.25% | motif file (matrix) |
| 3 | 0.792 | C T A G C T A G A T C G G A T C A C T G A C T G C T A G T A C G A G T C A G T C G C T A | 1e-39 | -90.555736 | 4.58% | 1.97% | motif file (matrix) |
